# Polyphenols from Root, Tubercles and Grains Cropped in Brazil: Chemical and Nutritional Characterization and Their Effects on Human Health and Diseases

**DOI:** 10.3390/nu9091044

**Published:** 2017-09-20

**Authors:** Diego dos Santos Baião, Cyntia Silva de Freitas, Laidson Paes Gomes, Davi da Silva, Anna Carolina N. T. F. Correa, Patricia Ribeiro Pereira, Eduardo Mere Del Aguila, Vania Margaret Flosi Paschoalin

**Affiliations:** Instituto de Química, Universidade Federal do Rio de Janeiro, Cidade Universitária Av Athos da Silveira Ramos 149, 21949-909 Rio de Janeiro (RJ), Brazil; diegobaiao20@hotmail.com (D.d.S.B.); cyntia.freitas@yahoo.com.br (C.S.d.F.); laidsonpaes@gmail.com (L.P.G.); daviuerj@gmail.com (D.d.S.); annac.correa@hotmail.com (A.C.N.T.F.C.); biopatbr@gmail.com (P.R.P.); emda@iq.ufrj.br (E.M.D.A.)

**Keywords:** beetroot, cassava, taro, cocoa nibs, soybeans and antioxidant activity

## Abstract

Throughout evolution, plants have developed the ability to produce secondary phenolic metabolites, which are important for their interactions with the environment, reproductive strategies and defense mechanisms. These (poly)phenolic compounds are a heterogeneous group of natural antioxidants found in vegetables, cereals and leguminous that exert beneficial and protective actions on human health, playing roles such as enzymatic reaction inhibitors and cofactors, toxic chemicals scavengers and biochemical reaction substrates, increasing the absorption of essential nutrients and selectively inhibiting deleterious intestinal bacteria. Polyphenols present in some commodity grains, such as soy and cocoa beans, as well as in other vegetables considered security foods for developing countries, including cassava, taro and beetroot, all of them cropped in Brazil, have been identified and quantified in order to point out their bioavailability and the adequate dietary intake to promote health. The effects of the flavonoid and non-flavonoid compounds present in these vegetables, their metabolism and their effects on preventing chronic and degenerative disorders like cancers, diabetes, osteoporosis, cardiovascular and neurological diseases are herein discussed based on recent epidemiological studies.

## 1. Introduction

Natural polyphenols are secondary metabolites found in vegetables and edible plants, cereals, fruit, seeds, oils and products manufactured from foods such as non-alcoholic and alcoholic beverages, like tea and red wine, and, mainly, cocoa products [1,2]. In plants, polyphenols are involved in the protective response to different stresses, including abiotic stresses, such as ultraviolet radiation where these compounds can impair oxidative stress and DNA damage, in injuries to the vegetal body where polyphenols stimulate the lignification process, contributing to healing, or in the defense against aggression by pathogens, where their concentrations may be increased after infection by virus, fungus, bacteria and even nematodes [3]. In foods, these compounds may contribute to bitterness, astringency, color, flavor, odor and oxidative stability [4]. 

Systematic reviews and meta-analysis studies have shown that dietary polyphenol intake may be associated with a decreased risk of chronic and degenerative diseases. 

After evaluating 22 prospective studies, Grosso et al., 2017 [5] concluded that the high consumption of total flavonoids decreased the risk of all-cause mortality. However, a 100-mg/day increment in flavonoid intake led to near to 5% decreased risk of all-cause and CVD (cardiovascular disease) mortality. Data from 18 human randomized controlled trials regarding flavonol supplementation were pooled and some heterogeneity was observed in the individual physiological responses of cardiometabolic biomarkers, such as blood lipids, blood pressure and plasma glucose [6]. Variability in the response of blood lipids to supplementation with flavonols was found, although significant reductions in total cholesterol, LDL (low-density lipoprotein) cholesterol and triacylglycerol, as well as a significant increase in HDL (high-density lipoprotein) cholesterol was a general effect among individuals. A significant reduction was also observed in fasting plasma glucose and in systolic and diastolic blood pressures. The reduction in CVD risk after flavonol supplementation was more pronounced in participants with diagnosed disease or dyslipidemia [6]. 

Cardioprotective effects were obtained for intakes of six classes of flavonoids, namely flavonols, anthocyanidins, proanthocyanidins, flavones, flavanones and flavan-3-ols [5,6,7]. Polyphenol dietary intake may be also associated with reduced risk for type 2 diabetes. A meta-analysis of 6 prospective cohorts involved 18,146 cases and 284,800 participants found that consumption of dietary total flavonoids is associated with a reduced risk of type 2 diabetes. Beneficial effects of polyphenol consumption were observed in the US population in men aged >40 years old people and in studies ≥20 years in duration [8]. Certainly, the reduction on diabetes risk contributes to the overall risk reduction of CVD.

It is estimated that at least 8000 polyphenols have been already described, considering natural, semi-synthetic or synthetic compounds, and this broad set of structures underlies the unpaired physico-chemical and biological properties displayed by each class of compounds. Polyphenols can be classified into main classes including phenolic acids, flavonoids, stilbenes and lignans. However, food matrices generally contain a complex mixture of those compounds, at variable concentrations, which may not be well characterized.

To review existing knowledge on selected polyphenol-rick foods characteristic of the Brazilian region, the bioactivity of polyphenols present in root and tubercle vegetables, such as taro, beetroot and cassava, and in grains, like soybean and cocoa, will be discussed. Some of these vegetables and grains are highly consumed commodities and considered valuable crops worldwide. The effects of these polyphenols will be explored, such as their role in preventing degenerative disorders like cancers, diabetes, osteoporosis, and cardiovascular and neurological diseases [9]. 

## 2. Polyphenol Structural Characterization and Classification

**Figure 1:**
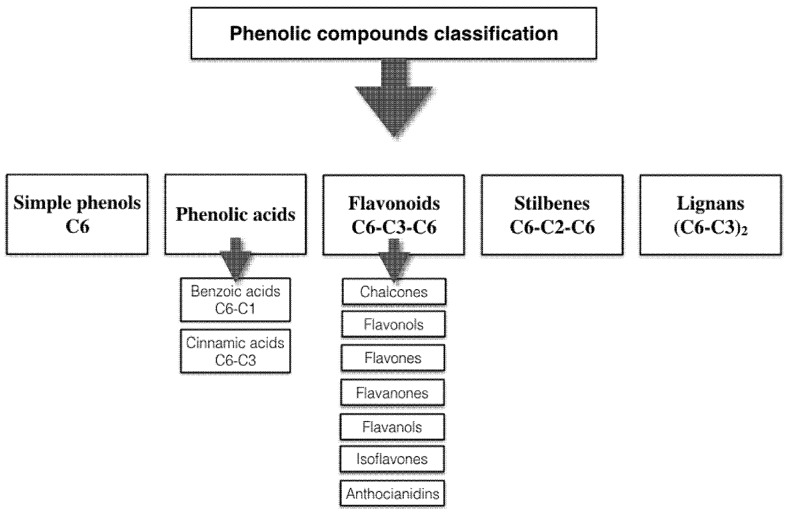
Schematic representation of the main classes and subclasses of phenolic compounds and their respective chemical structure backbone, where C6 corresponds to the aromatic ring and C1, C2 or C3 refers to side chains or intermediate chains.

Phenolic compounds are named when at least one hydroxyl group (–OH) is bound to one or more benzene aromatic rings (C_6_H_5_ or C6), forming the phenol structure (C_6_H_5_OH). If they exhibit a single aromatic ring, they are termed monophenolics or simple phenolics. As suggested by their nomenclature, polyphenols refer to molecules with two or more aromatic rings and at least one hydroxyl group attached to these rings, giving rise to a heterogeneous group of chemical compounds [10,11]. Based on the number of phenolic hydroxyl groups, the presence of specific functional groups attached to the benzene ring and the type of connection between rings, phenolic compounds can be divided into several classes [10] (Figure 1).

Since 1957, many attempts have been made to determine an adequate definition for the term polyphenol, however, none fits all the existing variety of polyphenols. In spite of this, a standard definition, which is a combination of the proposed definitions throughout the years, has been established, although still excluding simple phenolic compounds [11]. Based on this, the majority of scientific papers consider all phenolic compounds as a polyphenol, irrespective of the number of aromatic rings [10,12] based on the fact that they share similar properties and characteristics with those composed of multiple aromatic rings [13]. This tendency is also widespread in the agricultural, food and pharmaceutical industries, where the term polyphenols is preferred for commercial purposes, and includes any type of phenolic compounds [11]. The present review will not differ in this point, since it will discuss polyphenols as a unique group, including the simplest ones. 

**Figure 2:**
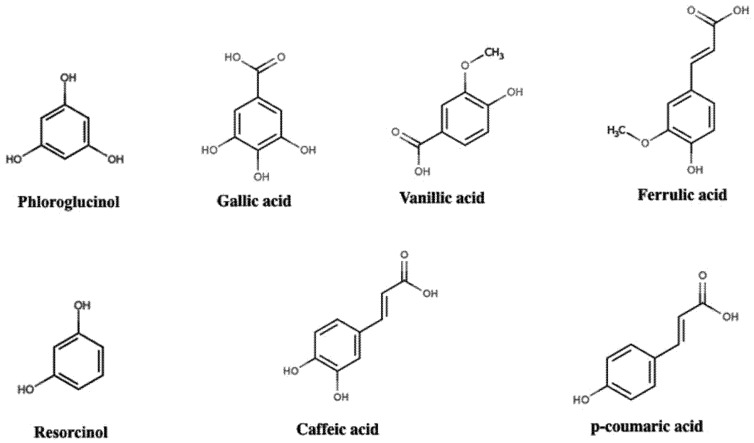
Chemical structures of phenolic acids, adapted from the database “Polyphenol content in foods” (http://phenol-explorer.eu). Gallic acid and vanillin acid belongs to benzoic acids subclass, characterized by a backbone composed of 7 carbons (C6-C1). Caffeic acid, ferric acid and p-coumaric acid represent the cinnamic acids subclass and exhibit a backbone composed of 9 carbons (C6-C3) [1].

The simplest phenolic compounds exhibit one phenol structure and up to three functional groups (R, R1 and R2) attached to the aromatic ring, but with the hydroxyl group as the main functional group. Argan (*Argania spinosa*) oil is a rich source of simple phenolic compounds, which are mainly represented by resorcinol, composed of two hydroxyl groups linked to the benzene ring. Phloroglucinol, containing an additional hydroxyl group, is another example of a simple phenolic compound, which has been extracted from *Eucalipitus kino*, *Acacia arabica* and marine algae (Figure 2) [10,14]. 

The phenolic acid class can also display the same short and simple structures, but with the additional presence of a carboxyl group (–COOH) attached to the aromatic ring, besides hydroxyl or methoxyl groups, which total up to three functional groups, similarly to simple phenols. This class is divided into two subclasses, benzoic acids and cinnamic acids, characterized by a backbone containing 7 (C6-C1) and 9 (C6-C3) carbon atoms, respectively [12,15]. Benzoic acids can be represented by gallic acid (R=R1=R2=OH) and vanillic acid (R=OH, R1=OCH_3_, R2=H) (Figure 2) [16]. 

**Figure 3:**
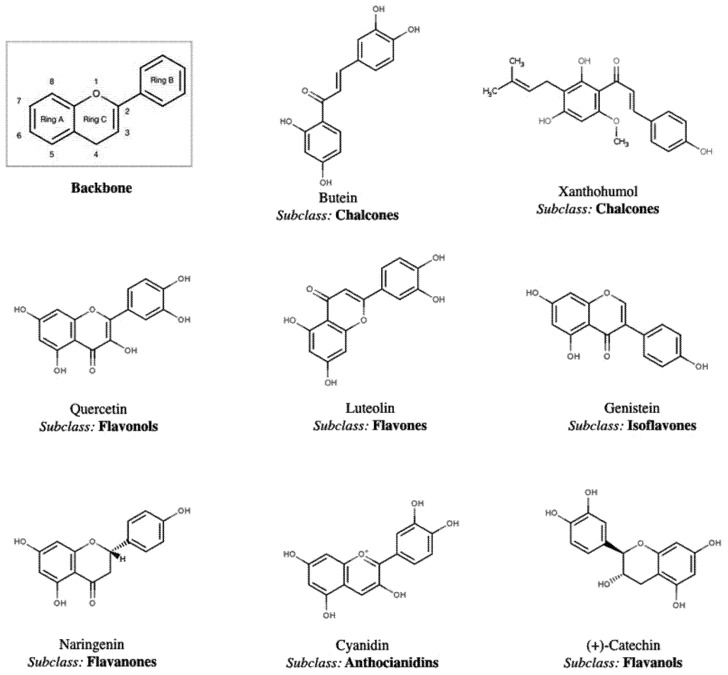
Representative chemical structures of the main subclasses and basic structure of the majority of flavonoids, adapted from the database “Polyphenol content in foods” (http://phenol-explorer.eu) [1].

The largest and most studied class of polyphenols is represented by flavonoids, which account for over 50% of phenolic compounds. They are composed of 15 carbon atoms that form two aromatic rings (A and B), connected by three carbons, giving rise to the typical C6-C3-C6 backbone (Figure 3). The three intermediate carbon atoms can assume different configurations, ranging from open to heterocyclic chains, condensed with the aromatic ring A. In this case, the intermediate chain is called ring C [10,15]. When ring C is arranged in the heterocyclic format, the basic phenylbenzopyran or chromane structure is generated, where benzopyran is represented by ring A (C6) fused to ring C (C3) and phenyl, by ring B (C6) [17]. Different configurations of the intermediate C3 chain and ring B will determine flavonoid classifications into their main subclasses, namely (i) chalcones; (ii) flavonols; (iii) flavones; (iv) isoflavones; (v) flavanones; (vi) anthocianidins and (vii) flavanols [12]. Several other types of substitutions (oxygenation, alkylation, glycosylation, alkylation and sulfation) in the flavonoid backbone are possible and are responsible for the high variety of more complex chemical compounds within this class [10,15]. Some flavonoids are restricted to only a few foodstuffs, such as isoflavones present in soy (about 1 mg of genistein and daidzein per g), flavonones present in citrus fruits (about 100–250 mg of hesperidin per L), flavanols present in some teas (about 200–300 mg of catechin, gallocatechin and their galloylated derivatives per g) and anthocyanins present in strawberries (4.5 mg per g of fresh fruit), beetroots (up to 19 μM trolox per mg) and red wine (26 mg per L) [18,19].

Chalcones are easily recognized among the flavonoid subclasses, since both aromatic rings are held together by an open C3 chain, while all the other six subclasses differ from each other according to the position where the ring B connects to the benzopyran portion. In most of these compounds, ring B is connected to the C2 of the heterocyclic ring C, but attachments to positions 3 and 4 can also occur (Figure 3). In some cases, chalcones are responsible for the yellow color in flowers, and are also present in fruits, such as apples, for example [12,15]. Except for the isoflavone subclass, where ring B connects to the carbon 3 of the benzopyran portion, the other compounds have their phenol portion attached to carbon 2. Isoflavones, heat sensitive compounds, are structurally similar to estrogen, being able to act as an estrogen mimetic. The richest sources of isoflavones are leguminous items, especially soybean (*Glycine max*) [20]. Flavones and flavonols exhibit a double bond linking carbons 2 and 3 of ring C, while flavonols display an additional hydroxyl group at position 3. Flavonol synthesis in plants is positively influenced by sunlight, and these compounds are easily found in the outer and aerial parts, while flavones accumulate in fruit skin but are not dependent on sunlight. Carbons 2 and 3 in flavanones and flavanols are not linked by unsaturation, but an oxygen atom is present at position 4 in flavanones and a hydroxyl group at position 3 in flavanols that differ from one another (Figure 3). Flavanones are typical constituents of citrus fruits, while flavanols are also known by their representative compound catechin, present mainly in green tea and chocolates [21,22]. 

Anthocyanidins differ from other polyphenols by positively charged oxygen, also called a pyrylium cation, in ring C (Figure 3). These molecules are usually associated to glycans and, in this case, are called anthocyanins. They are responsible for producing color in some plant parts, such as flowers, leaves and fruits, ranging from red to purple. Color production is dependent on pH, the presence of metal ions, type of attached sugar or acylester and anthocyanidin mixture. Two variations are possible in anthocyanidin chemical structure, including the presence of methyl groups at carbon 5 or 7 and the absence of a hydroxyl group in carbon 3 [21]. Rich sources of anthocyanidins include colorful fruits and vegetables, such as berries, grapes and red/purple vegetables [23].

**Figure 4:**
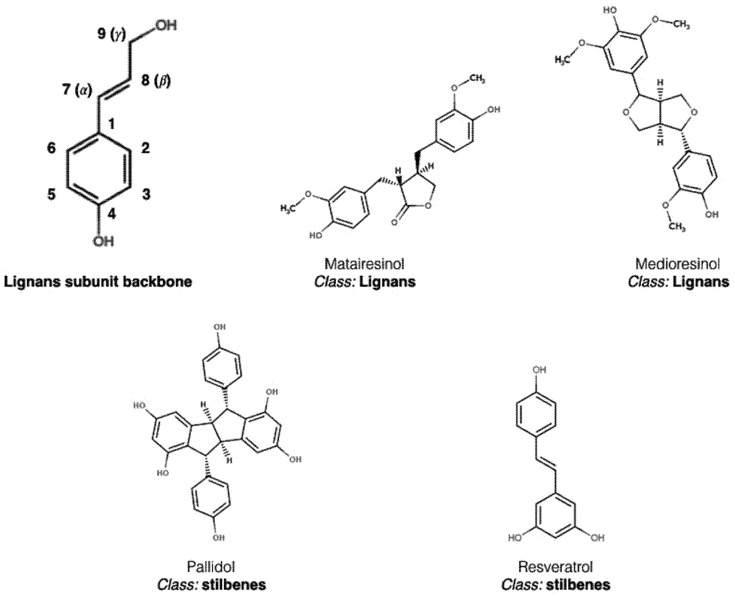
Chemical structures of the stilbene and lignan classes, adapted from the database “Polyphenol content in foods” (http://phenol-explorer.eu) [1].

Minor classes are represented by stilbenes and lignans, which, despite their importance to agro-food and pharmaceutical industries, are less widespread and found in few food products, mainly wines, some berry fruits and their juices. This class has a backbone quite similar to flavonoids, with the difference that both rings (A and B) are connected by a chain of two carbons (C6-C2-C6), which can occur in monomeric or oligomeric forms, and are represented by the widely known resveratrol, a typical wine constituent (Figure 4). The compounds generally derived from resveratrol belonging to class occur in their free form or associated with glycans [10,22]. Lignans originate from the dimerization of derivatives from C6-C3 compounds, such as p-coumaryl, conferyl and sinapyl alcohol, originating the typical lignan (C6-C3)_2_ subunit backbone [17]. In classical lignans, the dimer is stabilized by the interaction between at least two carbons 8, also named Cβ by some authors, from the side chain C3. Subunit connections involving carbons other than Cβ originate non-classical lignans, or neolignans. Oxygenation of at least one of the aromatic rings is also observed in this class [24]. Lignans are found in dietary fibers, especially flax seed and tea, which make them important compounds in the prevention of some types of cancer. This class is also known as phytoestrogens, since these compounds are converted into enterolactone and enterodiol after ingestion by humans, and then act in controlling sex hormone levels by inhibiting estrogen synthesis, which is directly related to the development and progression of cancer in endocrine-related reproductive tissues [25].

Many other phenolic compounds can also be found in plant-derived food, albeit in small amounts, and, for this reason are mostly not included in the general phenolic compoundclassification. However, some authors also include coumarins [17] and tannins into this classification. Tannins were the first phenol compounds to be reported and extensively studied by scientists. These molecules are ellagic or gallic acid polymers, or both, with a glucose molecule attached, and can also be found as a condensed form of two flavan-3-ol molecules. Considered anti-nutritional compounds, tannins have the ability to bind proteins of animal origin preventing putrefaction, which makes them powerful tools to convert animal skin into leather [17,22]. Coumarins, like cinnamic acids (Figure 2), exhibit the C6-C3 backbone, but the C3 chain is arranged as a heterocycle, formed by the presence of an oxygen atom, and can be represented by warfarin, a powerful anticoagulant compound [10].

In the last years, a significant number of studies have suggested that the extended consumption of vegetables or foods rich in polyphenols fulfill physiological needs and confer significant benefits to human health, such as combating oxidative stress, working as adjuvants in reducing the risk of developing chronic diseases, like cardiovascular conditions and protecting against the development of cancers, infections, aging, asthma and neurodegenerative diseases [26,27]. 

A significant number of studies, applying in vitro and in vivo approaches regarding he effects of polyphenol-rich vegetables have been conducted. Most of these human intervention studies have been performed concerning the dietary intake of an ingredient, whereas many in vitro studies have been performed regarding individual polyphenol food components [3,4,11,13]. 

## 3. Bioavailability and Dietary Polyphenol Intake

Some studies used as references concerning daily polyphenol intake have demonstrated high variability in polyphenol consumption. A comprehensive survey of the food occurrence of polyphenols must be undertaken using well-standardized methods, and the content of each polyphenolic compound should be expressed as the amount provided by a food serving. A very useful tool is the Phenol-Explorer database to estimate the content of polyphenol in different food matrices. However, polyphenol intake depends not only on individual food preferences, but also on cultural and climate factors, which may influence the dietary consumption of distinct populations.

The mean intake of 377.5 mg per day of total polyphenol (distributed in 284.8 mg/day of phenolic acids and 54.6 mg per day of flavonoids) for people living in the biggest metropolitan area in Brazil, São Paulo has been estimated. Coffee contributed to 70.5% of total polyphenols (flavanols and hydrocynnamic acids), citrus fruits, 4–6% and tropical fruits, 3–4% (flavonones and antocyanins). Intakes were higher in the elderly adults than in other adult groups (*p* < 0.001) and higher in individuals with lower educational levels [28]. In a subsequent study, where 620 elderly Brazilians participated in the survey, the average total polyphenol intake was of 1198.6 mg per day, distributed between phenolic acids (729.5 mg per day) and flavonoids (444.7 mg per day). The main dietary contributors for total polyphenols were coffee (45.8%) and beans (32.8%) [29]. The total polyphenol intake described for the elderly is similar to that estimated by a cohort study (1937 individuals (adults over the age of 18) of an urban population of Catania, Italy), where the dietary intake and major food sources of polyphenols in the Mediterranean healthy Eating, Aging and Lifestyles (MEAL) were evaluated. The mean polyphenol intake was of 663.7 mg per day; the most abundant classes were phenolic acids (362.7 mg per day) and flavonoids (258.7 mg per day), where the main dietary sources of total polyphenols were nuts, followed by tea and coffee, fruits, vegetables, chocolate, red wine and pasta [30].

Flavonol comsumption has been estimated at 20–25 mg per day and 35 mg per day in the USA, Denmar, Holland and Italy [31]. Europeans and Americans consume low amounts of isoflavones per day because of the very low consumption of soybeans in these countries. However, Asian isoflavones dietary ingestion is about 25–40 mg per day [32]. 

Studies concerning the bioavailability of polyphenols in several food matrices should be carried out to better understand the performances of these substances in the organism. The digestion, absorption and metabolization of each polyphenol differ, and there is frequently no relation between the polyphenol amount in food and the bioavailability in body. In the same way, the chemical structure of polyphenols in food matrices can be distinct from those found in human body fluids. For example, while aglycones are well absorbed in the small intestine, other polyphenols are well absorbed in other parts of the digestive tract. However, the majority of polyphenols arein the ester, glycoside and polymer forms and cannot be absorbed in their native form. These compounds must be hydrolyzed by enzymes from the colon microflora before being absorbed. For example, most glycoside forms resist acid hydrolysis in rat stomachs, although absorption in the gastric portion occurs for some flavonoids, such as quercetin, but not their glycosides [33]. In general, plasma concentrations vary according to the nature of the polyphenols and the food source [34,35]. 

During polyphenol absorption, chemical modifications, such as methylation, alkylation, sulfatation and glucuronidation, may take place in the small intestine and liver. Polyphenol methylation generally occurs at the C3 or C4 positions, and methylated polyphenols have been observed in plasma and urine [36]. In the liver, sulfo-transferase enzymes catalyze the transfer of a sulfate in the sulfation process that triggers metabolic activation [37]. The glucuronidation process occurs in the intestine and liver, where the highest conjugation rate is observed at the C3 position with a high efficiency rate. These chemical modifications restrict potential toxic effects and facilitate polyphenol biliary and urinary elimination. Extensively conjugated metabolites are preferentially eliminated in bile, while small conjugated metabolites are excreted through the urine. When polyphenols are secreted via the biliary route into the duodenum, bacterial enzymes act on these compounds causing their reasbsorption. In fact, the polyphenol forms that reach the blood and tissues may be distinct from the original forms found in foods. Thus, these chemical modifications add to the difficulties regarding the identification and evaluation of the biological activities of polyphenol metabolites [38]. 

## 4. Polyphenols and Their Cellular Effects

Phenolic compounds are related to a broad spectrum of medicinal properties with confirmed effects such as anti-allergic, anti-inflammatory, antibacterial, anti-thrombotic, vasodilators and, mainly, antioxidants [39]. Flavonoids, as other polyphenols, are important antioxidant compounds that can scavenge negative oxygen ions and free hydroxyl radicals in vivo [40]. 

Alongside the consumption of high amount of fruits and vegetables, that can impact human health [3], the beneficial effects derived from phenolic compounds found in these foods have been attributed to their antioxidant activity [41]. 

Phenolic acid benefits have also been exploited as cosmetic ingredients. Hydroxycinnamic acids and their derivatives have emerged as multifunctional ingredients for topical applications, since they display antioxidant, anti-collagenase, anti-inflammatory, antimicrobial and anti-tyrosinase activities, as well as ultraviolet (UV) protective effects, suggesting that they can be exploited as anti-aging and anti-inflammatory agents, preservatives and hyperpigmentation-correcting ingredients [42].

Commercially available ferulic and caffeic acids display anti-collagenase and photoprotection bioactivity. The use of 15–30 μM of ferulic acid and 3.75–30 μM caffeic acid promote the suppression of UVA-induced MMP-1 activity, offering protective activity to UVB-induced skin erythema [43].

Among the main vegetable products with high phenolic compound content, chocolate has been increasingly reported as a healthy and functional food, and the chocolate manufacturing industry has influenced the increase in the demand for this product [44].

## 5. Vegetables Popularly Consumed in Brazil as Polyphenol Sources

Vegetables are considered important sources of bioactive compounds, favoring human health and good organ function [45]. When the intake of foods rich in bioactive compounds is low, an increase in the production of reactive oxygen species (ROS) may occur, causing oxidative stress. Excess ROS may cause damage to DNA molecules, cell membranes, lipids and proteins [46]. This oxidative stress may lead to increased risks of developing non-communicable chronic diseases [47]. In addition, inadequate storage conditions and inappropriate culinary preparations may also adversely affect polyphenol content in different vegetables [48]. 

**Figure 5:**
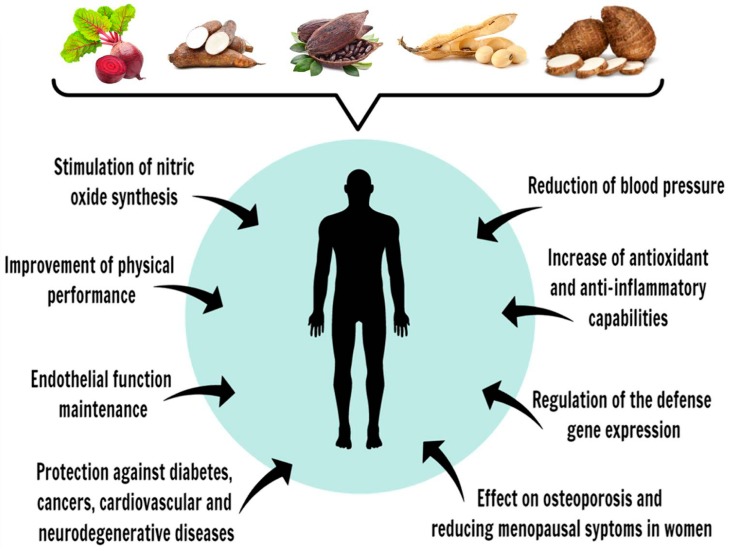
Polyphenolsfrom vegetables popularly consumed in Brazil—taro, beetroot, cassava, soybean and cocoa—and their effects on human health promotion and diseases.

Brazil is among the five largest food producers in the world, producing several different crops of edible plants, for both domestic consumption and exportation [49]. Some of these vegetables that display health properties have been studied following several research interests. This review reports both the composition and the content of phenolic compounds found in commercial vegetables commonly consumed by the Brazilian population, such as taro corms and leaves, cassava, beetroot, soybeans and cocoa nibs. The polyphenol typesand contents found in each food matrix are listed in Table 1, Figure 5, and in the subsequent sections of this review.

### 5.1. Beetroot (Beta vulgaris sp.)

Red beetroot has a high nutrition value (high sucrose content) and is considered a source of bioactive compounds, such as nitrate (NO_3_^−^), antioxidant substances and phenolic compounds, in addition to being a good source of dietary fiber, minerals (potassium, sodium, iron, copper, magnesium, calcium, phosphorus and zinc) and vitamins (retinol, ascorbic acid and B-complex) [66,67,68]. The red beetroot *Beta vulgaris* species is member of the Chenopodiaceae family, originated in regions of Europe and North Africa, where it is cultivated in mild to cold temperatures ranging from −10 to 20 °C. This plant species develops better in soils rich in organic matter and, therefore, the concentration of bioactive constituents depends on the plant development stage [69]. 

The antioxidant potential activity of red beetroot and its potential bioavailability in humans have been previously described [70]. The cited study reported that the most important betalain found in beetroot, betanin, is the most effective lipid peroxidation inhibitor, while other important compounds found in beetroot are polyphenols. A great controversy regardingpolyphenol contents in red beetroot exists, as in other vegetables. Indeed, in general, polyphenol content in vegetables is quite variable and can be characteristic for each genetic variety, but several factors like pedoclimatic (soil type, sun exposure and rainfall) or agronomic (culture in greenhouses or fields, biological culture and hydroponic culture) environmental factors, ripeness at the time of harvest, processing, and storage can affect the concentration of polyphenolic compounds in vegetables [71,72]. The total polyphenol content in beetroot has been reported as ranging from 720 to 3764 mg per kg [73,74]. 

A particular study determined the antioxidant potential, identified and quantified phenolic compounds in a juice prepared from beetroot, including gallic (ranging 10.8 to 30.4 mg L^−1^), syringic (ranging 0.67 to 3.54 mg L^−1^), caffeic (ranging 3.03 to 10.3 mg L^−1^) and ferullic (ranging 0.25 to 1.24 mg L^−1^) acids [44]. Considering a beetroot juice shot, the total polyphenol content ranged from 977.2 ± 5.2 to 1450.3 ± 42.1 mg and 3189.1 ± 77.3 to 1527.1 ± 18.0 mg of gallic acid equivalents/L before and after in vitro digestion, respectively [69]. Beetroot also displays antioxidant capacity estimated from ORAC and FRAP measurements, ranging from 23.9 to 37.9 mmol L^−1^ and 17.4 to 37.1 mmol L^−1^, respectively, but the antioxidant capacity of beetroot juice differs among the beetroot varieties and is dependent on the total polyphenol content of each genetic variety analyzed [50]. Besides phenolic amides, such as *N-trans-*feruloyltyramine and *N*-*trans*-feruloylhomovanillylamine, and flavonoids such as betagarin, betavulgarin, cochliophilin A and dihydroisorhamnetin, several betalains, including vulgaxanthins I and II, betanin and isobetanin, have also been detected and quantified in red beetroot [75].

Although whole beetroot has become the most common formulation used for dietary administration, other beetroot formulations have been developed, such as beetroot juice and chips powder, and their antioxidant power and phenolic content has been evaluated [76,77]. Since foods in gel form might be the most effective formulation, due to the high concentration of bioactive compounds in a reduced volume, a beetroot gel has also been developed [78] from a concentrated beetroot juice and, as expected, showed higher levels of total phenolic compounds and antioxidant capacity, as well as other bioactive compounds [78]. The new beet formulation was shown to be easy to carry, ingest and tolerate, and easy to store at room temperature [79].

### 5.2. Cassava (Manihot esculenta)

Cassava is a tuberous woody shrub belonging to the Euphorbiaceae (spurge) family. This all-season crop, originated in the New World, but, is currently a staple food and animal feed in tropical and subtropical Africa, Asia and Latin America [80]. It is known as “tapioca” in Asian countries, as “mandioca”, “aipim”, “castelinha” and “macaxeira” in Brazil, as “yuca” in Spanish-speaking countries of Latin America, and as “manioc” in French-speaking countries in Africa [81].

Brazil is the second largest cassava producer in the world, with a production of 49% concentrated in Northeastern Brazil. In this region, skewed land distribution and semi-arid conditions go hand in hand with high poverty levels. According to the Brazilian Institute of Geography and Statistics [49], the cassava-planted area in Brazil is of 2.035 million hectares, with a production of 23.242 million tons and an average yield of about 12.984 tons per ha [49]. Brazil’s contribution to the world’s cassava production is of around 16.2% and, for Latin America, of 77% [82]. In Brazil, two distinct cassava crops are available: *bitter (mandioca)* and *sweet (aipim)*. The bitter type is the main crop, with high cyanogenic potential, and should be processed prior to consumption. The main product is coarse, toasted flour used to complement many other dishes, enriching carbohydrate food content. Brazil has established a research program at the National Cassava and Fruit Research Center (CNPMF) that holds the world largest collection of cassava germplasm [49]. 

Cassava starch can be spontaneously fermented and sundried, and the resulting sour cassava starch (“polvilho azedo”, in Brazil or “almidón agrio”, in Colombia) is used for the production of gluten-free breads and biscuits that are very popular in some South American countries, and is considered the main source of dietary food energy for the majority of people living in the lowland tropics, and much of the subhumid tropics of West and Central Africa [83,84,85]. Certain cassava flour and starch properties, such as physical, chemical, physicochemical, pasting, and thermal parameters are important for its use in the food industry [84,86].

The phyto-nutritional status of cassava crops is influenced by several factors, such as plant genotypic background, climatic conditions, cultural practices and the use of inorganic fertilizers, causing significant variations in vitamin concentrations, antioxidant capacity and phenolic content [87]. 

Cassava roots can be processed into a wide variety of granules, pastes or flour, or consumed freshly boiled or raw. Processing can affect the nutritional value of cassava roots through molecule modifications and nutrient losses. Analyses performed on nutrient retention for each edible cassava product indicate that raw and boiled cassava root maintain the majority of their high-value nutrients, except for riboflavin and iron. Although raw cassava roots contain significant vitamin C amounts, they are very sensitive to heat and this nutrient can easily leach into water. Therefore, almost all of the processing techniques seriously affect raw cassava root content [88].

Cassava plant extracts contain a wide range of phenolic compounds including flavonoids, tannins, terpenoids, glycosides and alkaloids [86,89]. Cassava roots easily deteriorate during storage soon after their harvest and when, damaged by cutting or fungal infection, phenolic compounds, such as scopolin, scopoletin, and diterpenoids, are accumulated in the injured or infected regions [56,90,91]. The raw cassava tuber also contains alkaloids, flavonoids, tannins, reducing sugars and anthocyanosides, but does not contain cardiac glycosides, anthraquinone, phlobatinnins or saponins [88]. 

Coumarins are by far the main cassava component, while other polyphenols, like catechins, are present in minor concentrations and have been proven present in experimentally injured root parenchymas [92,93]. Among coumarins, catechin gallate, gallocatechin and flavone 3-glycosides (rutin and kaempferol 3-rutinoside) are observed, which have been suggested as displaying cardiovascular health benefits [56]. 

In addition, cassava also contains stilbenes. Two of these compounds, *trans*-3,3′,5,5′-tetrahydroxy-4′-methoxystilbene and *trans*-3,4′,5-tetraxydroxystilbene (resveratrol), have been identified to date [59]. In addition, some unique compounds with a spiro configuration have also been isolated and characterized, such as the spiro biflavonoid larixinol, biosynthesized by combining two C15 units from a flavonoid origin and a number of novel spiro-structures, which were given the common names of yuccaols A–E [94,95], and a stilbenic portion closely related to resveratrol. The content of these stilbenes is described in Table 1 and have been compared to reverastrol content in the same matrix [95]. 

Cassava also contains several antioxidants, such as alpha- carotene and vitamin A [96,97]. In addition, cassava roots are both consumed fresh and stored for several days. An evaluation of the bioactive compounds present in this vegetable was carried out and reported the negative effects of its rapid deterioration [90,93,98]. Cassava tubers have been shown to contain polyphenol oxidase isozymes, which can oxidize monophenols into o-diphenols and/or diphenols into their corresponding o-quinones [99]. Cassava leaves present great potential for human and animal consumption as a green vegetable, in replacement of conventional protein sources; although it is important to note that cassava leaves contain major anti-nutrients, such as hydrocyanic acid (HCN), tannic acid and phytic acid. Cassava leaves can provide proteins and vitamins A, B and C, as well as several minerals, including Mg, Fe, Zn and Mn [100,101]. In addition, anthocyanidins (cyanidin and delphinidin) [102] and flavonoids (quercetin, rutin and kaempferol) have also been identified in this cassava portion [103,104,105]. Tannins have also been reported, and their content in cassava leaves increases with plant maturity [106]. Shredding and sun-drying processing can reduce cyanide levels of cassava leaves, and may be associated with other methods in order to eliminate anti-nutrient compounds [96].

The selection of the solvent system significantly influences the recovery of each phenolic compound from cassava extracts. Phenolic compound content after an ethanol/acetone extraction was very similar for distinct cassava cultivars: gallic acid, 330 ± 4.5 mg kg^−1^; gallocatechin, 91.0 ± 2.7 mg kg^−1^; catechin, 15.6 ± 1.4 mg kg^−1^ and chlorogenic acid, 10.5 ± 3.32 mg kg^−1^ [53]. The anthocyanin content from cassava leaf stalks ranged from 44 mg g^−1^ in an acidified methanol extract, to 15 mg g^−1^ after acetone extraction. Antioxidant anthocyanin properties arise from their high reactivity as hydrogen or electron donors, and from the ability of the polyphenol-derived radicals to stabilize and delocalize unpaired electrons, as well as from their ability to chelate transition metal ions [107]. In addition, cassava flavonoid extracts display different health benefits [88,108,109]. In one study, ten antioxidant compounds (coniferaldehyde, isovanillin, 6-deoxyjacareubin, scopoletin, syringaldehyde, pinoresinol, *p*-coumaric acid, ficusol, balanophonin and ethamivan) were isolated and identified from cassava stems by an activity-guided isolation and were found to have DPPH scavenging capacity and ABTS free radical scavenging ability [58,110].

### 5.3. Cocoa Beans (Theobroma cacao) and Cocoa-Based Products

The fruit of the cocoa tree belonging to the Sterculiaceae family is a pod originated from Central America, widely cultivated by the Mayan civilization [111,112]. Cocoa is an important manufacturing plant with a significant influence on the economy of several countries [113] and is also a popular drink crop around the world, after coffee and tea. It is also widely used as the main ingredient in chocolate [114]. Chocolate is obtained from the fermented, roasted and milled seeds of the cocoa fruit by blending chocolate liquor with other ingredients, such as cocoa butter, sucrose, condensed milk or milk proteins, nuts, cereals or other ingredients [115].

Cocoa seeds are subdivided into cotyledon and embryo, protected by a pulp and a seed coat. They are basically constituted of 54% fat, 1.5% theobromine and caffeine, and polyphenol content represents 12 to 20% of the dry weight of the whole seed [116]. Polyphenol compounds are stored in the pigment cells of the cotyledons and are responsible for imparting bitterness and astringency to the cocoa fruit [117]. The natural microbiota involved in cocoa seed fermentation has a significant influence on polyphenol diffusion, but at the same time, is responsible for reducing almost 20% of the amount of these compounds in the final product [118]. Due to the high amount of polyphenols in cocoa, the number of studies demonstrating beneficial health effects of moderate consumption of phenolic compounds from cocoa products has increased. Improvementsin endothelial function, blood pressure, and cholesterol levels support potential benefits on cardiovascular function [119,120].

Spontaneous fermentation is a critical step for the development of chocolate flavor and taste, due to the generation of aroma precursors and alteration of the phenolic content of cocoa beans [121]. Bacteria and yeast consortia act on cocoa seeds during the fermentation process, producing indispensable metabolites, influenced by pH, temperature and oxygen availability [122]. During fermentation, the microbiota generates metabolites that influence flavor and may determine the quality of chocolate and cocoa-based products. During the fermentation process the production of lactic acid, acetic acid, lipids and proteins occurs, as well as of phenolic compounds that exert beneficial effects on human health.

The influence of phenolic compounds on chocolate flavor and color has been demonstrated in many studies, through reactions between polyphenols, sugar and amino acids, while alkaloids contribute to the bitterness of the fermented beans. Many studies have estimated phenolic compound contents during the fermentation process, where the highest total phenolic content was found after 24 h of fermentation, but decreased with longer fermentation times [123]. Polyphenols tend to diffuse out from the pigment cell of the cotyledons at the beginning of the fermentation process and undergo oxidation and complexation, producing mostly insoluble tannins. Polyphenol oxidases convert polyphenols into quinones, which then complex with proteins and peptides [118].

Of the several phenolic compounds identified and measured in cocoa beans and their by-products, such as cocoa liquor, cocoa powder and dark chocolate, the main groups are catechins or flavan-3-ols (37%), procyanidins (58%) and anthocyanins (4%) [115]. The main catechins are (−)-epicatechin, present in up to 35%, and, in smaller amounts, (+)-catechin, as well as traces of (+)-galloctechin and (−)-epigallocatechin. The anthocyanin fraction consists mainly of cyanidin-3-α-l-arabinosid and cyanidin-3-β-d-galactosid. Procyanidins comprise mostly flavan-3-4-diols, that are 4-8- or 4-6-bound to condensed dimers (procyanidin B1 dimers (166 mg kg^−1^) and procyanidin B2 (921 mg kg^−1^), trimers or oligomers with epicatechin (3.28 g kg^−1^) as the main extension sub-unit [124]. In addition, cocoa also contains other polyphenols in minor amounts, such as quercetin (25 mg kg^−1^), quercetin-3-*O*-glucoside (110 mg kg^−1^) (isoquercitrin), quercetin-3-*O*-galactoside (90 mg kg^−1^) (hyperoside), quercetin-3-*O*-arabinoside (165 mg kg^−1^), as well as the following flavones: apigenin (5 mg kg^−1^), apigenin-8-*C*-glucoside (4 mg kg^−1^) (vitexin), apigenin-6-*C*-glucoside (4 mg kg^−1^) (isovitexin), luteolin (5 mg kg^−1^), luteolin-7-*O*-glucoside (12 mg kg^−1^) and methylxanthines, mainly theobromine, as well as caffeine in small amounts [125].

Many studies have demonstrated the beneficial effects of chocolate on the cardiovascular system [126,127], where polyphenol have been shown to attenuate intracellular pro-inflammatory reactivity [128]. In obese and overweight individuals, dark chocolate-polyphenols were equally effective in reducing fasting blood glucose levels, systolic and diastolic blood pressures, while a decrease in free urinary cortisone levels in both groups was also observed [129]. Cocoa flavonols are effective in reducing blood pressure, insulin resistance, lipid peroxidation and improving cognitive function in mildly impaired elderly subjects [130]. High-quality dark chocolate is also the richest in theobromine and caffeine, which are vasodilator, diuretic and heart stimulant compounds [122].

### 5.4. Soybeans (Glycine max)

Soy grain is a polyphenol-rich legume belonging to the oilseed family, and its production is the highest among the grain legumes in the world [131]. The worldwide interest in this grain is due to the versatile usage of its derived products for bothhuman and animal consumption, which confers a high economic value to this grain in the national and international markets. Brazil is among the largest soybean producers in the world, since this grain legume is grown in several regions of the country [132].

Soybean is widely accepted as a healthy food, due to its nutritional composition and pharmacological effects, which have been attributed to the presence of several phenolic compounds. In this grain, polyphenols confer color, taste and sensory properties, such as sweetness, bitterness and astringency [4,133]. The concentration of polyphenols in soybeans is influenced by many factors, such as cultivar type, agricultural period, planting site, soil nutrition and shelf-life [64].

Soybean is rarely consumed simply by cooking the beans. Traditionally, it is processed to generate grain-derived products or ingredients for the food industry. Several soybean-derivatives are obtained by fermentation, such as tempeh and doulchi, or by coagulation only, such as several types of tofu [133]. In addition, soybeans can be used in the production of isolated proteins or hydrolyzed vegetable protein, which in turn are used as ingredients in meat and bakery products, beverages, soups and several other food products [134,135]. In these cases, soy proteins may confer texture, water retention and gelification, while increasing the polyphenol content of the processed food.

Soybeans contain isoflavones, anthocyanins, phytic acids, saponins, phenolic acids, hydroxybenzoic and hydroxycinnamic acids, isoflavonoids and anthocyanins [65]. Isoflavones possess high antioxidant activity and metal-ion chelating properties, but their major importance is their role as phytoestrogens, binding to estrogen receptors, and causing an estrogenic or anti-estrogenic effects according to the type of estrogen receptor. Their intake has been associated with a decreased risk of hormone-related cancers [133].

Soybean phytoestrogens present low molecular masses and occur mainly in 4 chemical forms, malonyl-β-glucosides (70–80%), acetyl-β-glucosides (5%), β-glucosides (25%) and aglycones (2%), structurally similar to estrogen, estradiol-17β, and are able to activate or block estrogen effects, depending on the type of receptor, as mentioned previously [64,65,136]. 

The most investigated isoflavones are the aglycones daidzein and genistein, because they present antiproliferative activity against human breast cancer cell lineages by reducing the expression of estrogen receptors ERα and c-erbB-2 [137]. Phytoestrogens can bring many other health benefits besides the decreased risks of hormone-related cancers [138]. These include the prevention of vascular diseases through the inhibition of in vitro oxidation of the low-density lipoprotein involved in the pathogenesis of atherosclerosis [139], hypercolesteremia [140], osteoporosis [141] and menopausal symptoms [142].

Daidzein is mainly metabolized to equol and O-desmethylangolensin (O-DMA) by the human gut microflora, but both show strong biological activity [143]. In addition to isoflavones, black soybean possesses anthocyanins located in the soy seed coats, conferring the dark grain pigmentation [144]. The main anthocyanins found in black soybeans are cyanidin-3-*O*-glucoside, delphinidine-3-*O*-glucoside, and petunidin-3-*O*-glucoside [145], that display several biological activities, like antioxidant, anti-inflammatory, nephroprotective, antidiabectic, anticancer, anti-infertility, anti-obesity, anti-arthritic, neuroprotective, antihyperlipidemic and anti-cataract activity, as well as healing properties [63].

### 5.5. Taro (Colocasia esculenta)

*C. esculenta*, popularly known as taro, is an herbaceous plant native to India that belongs to Araceae family and is cultivated in both tropical and subtropical regions [146]. *C. esculenta* is capable of developing under conditions considered adverse to other species, such as poor soils, long raining periods, shading and other climatic stresses. This rustic plant displays low-cost crop production, with a high yield per unit area, is easy to maintain and resistant to pests and diseases [147,148].

Its edible corm is the main reason why this species is considered the 14th cultivated vegetable/staple around the world [149]. Taro contains substantial starch and fiber contents that can supply energy and satiate consumers, and is considered an essential food crop for millions of people worldwide [150]. Just not only is the corm edible, but taro leafs, petioles, inflorescences are also consumed, as well as taro derived-products, some of them industrialized, that have become outstanding and traditional foods worldwide. In order to add value and maintain quality, post-harvested technologies are usually applied to taro food and products, such as boiling, roasting, baking, frying in oil, pasting, milling and pounding [150,151], manufacturing alternative meals such as baby food, taro flakes, taro bread, dried taro chips, taro flour, noodles and even alcoholic beverages. Petioles are usually cooked and leaves are used in sauces and stews, purees or soups, while inflorescences are consumed cooked or fried [87]. 

Taro also shows other applications, not only in food products, but also for industrial purposes, including animal feed, insecticides, biodegradable products, and as an alcohol matrix producer [146]. Apart from its nutritional value, taro has also been investigated for its biological properties, due to the presence of a diverse range of biologically active phyto constituents present in the plant, such as flavonoids, alkaloids, sterols, tannins, phytates, glycosides and other micronutrients [151,152]. 

The well-known benefits promoted by polyphenol compounds, such as anti-inflammatory, antioxidant, antiallergic, hepatoprotective and antiviral activities, coincide with the therapeutic properties exerted by taro [153].

Forty-one phenolic compounds in taro leaves extracts have been identified and the main compounds are flavones di-*C*-glycosides, comprising around 84 to 87% of the total phenolic compounds in this matrix [87,154]. Tannins, flavonoids, triterpenoids and sterols have also been described in two distinct varieties of the plant, the “giant white” and “red” taros, during the same development stage. The phenolic content of both varieties was ca. 9 g kg^−1^ but differences in the type of compounds reinforced the diversity and different amounts of phenolic compounds among *C. esculenta* cultivars [155].

A recent study [156] addressed the total phenolic and flavonoid composition of taro corm, and observed that flavonoids are present at around one quarter of the total phenolic content, contradicting previous studies [157] that reported significantly higher differences in the phenolic content of taro corm. The main flavonoid found in taro corm listed in the USDA 2013 database is quercetin, at an average of 28.7 mg kg^−1^ of the edible taro corm portion [158]. Anthoncyanins have also been identified in higher amounts in corm skin (16 mg) when compared to both corm and petioles (3.29 mg in both vegetable parts) [159].

As mentioned previously, taro can be processed in different forms and prepared by various methods, while the concentration and availability of bioactive constituents can be modified during tuber processing by boiling and frying [158]. Flavonoid and alkaloid content in taro do not decrease when compared to the raw form following boiling, unlike frying. On the other hand, tannin contents significantly increased during boiling and frying processes. Apparently, each processing method can generate new bonds between taro phytochemicals and cell structures, leading to higher or lower phenolic cleavages. 

The industrial processing of taro into powder, noodles and cookies may modify its phytochemical contents. Therefore, a study was conducted to analyze the composition of raw taro, when processed into powder, noodles and cookies, and determine the effects of processing taro [160]. Results showed that, in general, taro powder increased phenol, tannin, flavonoid and saponin contents, whereas all these polyphenols are decreased in noodles and cookies, and tannins, in particular, are no longer detected. Cell disruption and decompartmentalization of phenolic compounds occurs when taro noodles and cookies are exposed to high temperatures for a long period of time, promoting polyphenol oxidase denaturation. Peeling, cutting and slicing taro also induces a quick enzymatic oxidation that helps to reduce the levels of these natural antioxidants and may cause negative effects on the final food products [160].

## 6. Dietary Intake of Selected Plants or Their Polyphenols and Human Health in Diseases: Intracellular Targets and Molecular Mechanisms

In the last decade, there has been much interest in the potential health benefits of dietary plant polyphenols, mainly as antioxidants. Although the mechanisms of protective polyphenol effects on degenerative diseases are not yet fully understood, several studies strongly suggest that long-term consumption of diets rich in plant polyphenols offers protection against different diseases. The phenolic groups within the structure of polyphenol molecules can accept an electron and thus form a stable phenoxyl radical, disrupting chain oxidation reactions in cellular components and, thereby, increasing plasma antioxidant capacity. Indeed, after polyphenol-rich food or beverage consumption, plasma antioxidant capacity increases significantly [18].

The antioxidant effect by which polyphenols exert theirbeneficial properties appears to involve their interaction with cellular signaling pathways and related machinery that mediate cell function under both normal and pathological conditions [161]. The signaling pathways for chronic and degenerative diseases should have their own intermediates and determining how polyphenols interact with those intermediate from the signaling pathways is the challenge to understand the multiple protective effects of those antioxidants. Nuclear factor kappa B, endothelial nitric oxide synthase and angiotensin converting enzyme have been recently identified, which may partly explain potential beneficial cardiovascular effects of polyphenols [162,163].

The following table lists selected biological effects related to the consumption of the electable plants of this study (taro, cocoa, cassava, soy and beetroot) or their polyphenols, described in a context of relevance to human health (Table 2).

It is evident that polyphenol compounds should be sufficiently absorbed, crossing the intestinal barrier and reaching micromolar concentrations in the bloodstream, where they have been shown to present biological effects [9,18]. However, most studies were not conducted in vivo, but in vitro, and did not take in account metabolic and bioavailability factors, while the reported observations do not necessarily occur in vivo. In general, polyphenol concentrations must range from 0.1 to 100 μmol L^−1^ in order to observe biological in vitroeffects. Taking into consideration that polyphenol physiological concentrations are less than 10 μmol L^−1^; the effects of polyphenols assayed in vitro using concentrations above 10 μmol L^−1^ may not be valid. In addition, the polyphenol forms that appear in the blood may be different from those found in foods, due to the extensive metabolism suffered by these compounds in the human organism, as mentioned previously. 

The organism is in a state of redox equilibrium in basal metabolic conditions, presenting a balance between oxidant and antioxidant agents. At low concentrations, oxidant agents originating from the cellular metabolism, such as reactive oxygen and nitrogen species (RONS) play important roles in several cellular and biochemical processes, including gene expression, cell proliferation, apoptosis and muscle contraction [172]. However, overexposure of a cell to RONS (as happens through exposure to infectious pathogens during metabolic and cardiovascular diseases, degenerative diseases, inflammatory processes or even in some physiological conditions, such as aging, can overwhelm the oxidant state, leaving DNA, carbohydrate, protein and lipid structures susceptible to oxidation and functional deficiencies, or, in other words, causing an imbalance in redox homeostasis and promoting oxidative stress [173].

LDL oxidation is considered one of the main mechanisms in atherosclerosis development, a chronic inflammatory disease that develops in lesion-prone regions of medium-sized arteries and can lead to the development of pathological conditions, which may result in death [38]. The total antioxidant activity before and after in vitro digestion procedure with simulated gastric and duodenal phases of beetroot juice shots [75] for example, has been evaluated, where an approximate 3-fold increase in total antioxidant capacity was observed, evaluated by ferric reducing antioxidant power (FRAP), after the gastric phase (33,731.42 ± 298.57 μmol L^−1^), with a marked decrease after the duodenal phase (24,861.42 ± 301.42 μmol L^−1^). The authors reported that the high antioxidant capacity of the beetroot juice shot was attributed to its polyphenol contents. The antioxidant capacity of 4880 ± 68.6 mg of GAE L^−1^ polyphenol content was reduced to 3188.6 ± 77.14 mg GAE L^−1^ following the gastric phase digestion, but still at 2/3 of the initial antioxidant level.

Cocoa polyphenol compounds display anti-inflammatory antioxidant and anti-atherogenic effects that promote several cardiovascular events. As expected for polyphenol, those present in cocoa polyphenols increase HDL-c (HDL-cholesterol), reduce LDL oxidation, inhibit platelet aggregation and decrease vascular cell adhesion, improving endothelial function and reducing blood pressure. But can also modulate intestinal inflammation through the reduction of neutrophil infiltration and expression of different transcription factors, which leads to decreases in the production of proinflammatory enzymes and cytokines.They also have antiproliferative, antimutagenic, and chemoprotective effects, in addition to their anticariogenic effects [174,175].

Yuccaols (A, B, C) are phenolic constituents isolated from *Yucca schidigera* barkm characterized by unusual spirostructures made up of a C15 unit and a stilbenic portion closely related to resveratrol, and are associated with the prevention of the platelet aggregation. This polyphenol inhibits cyclooxigenase 1 (COX 1) activity, reducing the synthesis of thromboxane A2 (TXA_2_), a vasoconstrictor and platelet aggregation inducer.The anti-inflammatory properties attributed to Yucca schidigera can be ascribed to both resveratrol and Yuccaols and provide the first evidences of the anti-tumor and anti-invasive properties of these novel phenolic compounds [176].

On the other hand, isoflavone intervention studies are perhaps the more advanced of polyphenol effects in human beings. Several studies have demonstrated protective isoflavone effects on lipid peroxidation and increased LDL resistance to oxidation. These studies have also shown effects on bone biomarkers, such as increased serum concentrations of bone-specific alkaline phosphatase and osteocalcin [165,177]. Bone metabolism and bone mineral density (BMD) were evaluated in 90 healthy post-menopausal women following the intake of the phytoestrogen genistein. Genistein prevented bone loss influenced by estrogen-deficiency without exerting negative effects on the uterus and breast. Furthermore, a decrease in bone resorption markers and enhanced bone formation parameters were observed. Genistein may exert these aforementioned beneficial effects on bones by inducing their apoptosis through the Ca^2+^ signaling pathway, and can also act directly by suppressing osteoclasts, decreasing the amount of these bone cells. This suppressing effect on osteoclasts may occur, at least in part, by the inhibition of protein kinase and the activation of protein tyrosine phosphatase caused by genistein. Furthermore, genistein significantly stimulates thymidine incorporation and increases cell numbers in human vertebrae–derived bone cells [165,177].

Atherosclerosis, a chronic inflammatory disease that develops in lesion-prone regions of medium-sized arteries, may be clinically silent for decades before becoming active and producing pathological conditions (acute myocardial infarction, unstable angina or sudden cardiac death). The effects of 17 days of a diet enriched in soy isoflavones containing 21.2 mg of daidzein and 34.8 mg of genistein were investigated on the in vivo biomarkers of lipid peroxidation and LDL resistance to oxidation, in 24 healthy volunteers (19 women and 5 men). The plasma concentration of 8-epi-prostaglandin F(2)(alpha) was decreased after the soy isoflavones treatment concomitant with the lag time extension for copper-ion-induced LDL oxidation [166].

The polyphenols may exert protective effects on human cancer cell lines, reducing the number or the growth of tumorigenic cells [178]. Polyphenols such as catechins, isoflavones, quercetin, flavanones, lignans, curcumin and resveratrol have been tested and all of them showed protective benefic effects in some tumor models although their action mechanisms are distinct [179]. Some chemoprevention mechanisms of these above mentioned polyphenols are changes in cellular signaling, induction enzymes detoxification, antiestrogenic activity, antiproliferation, anti-inflammatory activity, regulation of the host immune system, prevention of oxidation and induction of cell cycle arrest or apoptosis [175].

A study evaluated the effects of 1-month consumption of soy-derivative foods rich in isoflavonoid phytoestrogens (about 86 mg isoflavones) on LDL oxidation and sex hormone receptor activity in 31 hyperlipidemic subjects [180]. The authors observed a decrease in both oxidized LDL measured as conjugated dienes in the LDL fraction and the ratio of conjugated dienes to LDL cholesterol after consumption of soy-derivative foods rich in isoflavonoids. However, a non-significant decrease was detected in estrogenic and androgenic hormone activities, evaluated by estrogen and androgen (dihydrotestosterone) hormones after isoflavonoids-rich food intake. This result is important because it has already proven by scientific community that a massive increased estrogen levels tend to stimulate uncontrolled cell growth of breast epithelium, contributing to the development of breast cancer [181]. However, in the study mentioned above [180], no significant difference was detected in ex vivo estrogenic activities in urine samples of women after intake of high-isoflavones foods. Additionally, the reduction of the levels of circulating oxidized LDL was observed. Soybeans consumption may reduce cardiovascular disease risk without increasing the risk for hormone-dependent cancers, but confirming a tendency for the reduction of in ex vivo sex hormone activity. Soy-isoflavones ingestion could be administered in association with sex hormone blocking agents to treat and possibly prevent hormone-dependent cancers [181].

As mentioned before, catechin is a phenolic compound found at high concentrations in dark chocolate. Due to the widespread non-galloylated forms, it is difficult to estimate catechins during intervention studies. Catechins display many in vitro and in vivo effects. They can increase plasmatic antioxidant activity, the resistance of LDL to oxidation, raise ascorbate concentrations, decrease plasma lipid peroxide and malondialdehyde concentrations, and reduce non-heme iron absorption [182,183]. Several epidemiologic studies have demonstrated the beneficial association between cocoa intake and lower cardiovascular disease mortality [184,185], cardiovascular risk factors, such as lipid profile, insulin resistance and vascular dilatation [186,187,188].

The ingestion of increasing amounts of cocoa powder (13, 19.5 and 26 g day^−1^) for 4 weeks altered LDL and oxidized LDL cholesterol concentrations in the plasma of normocholesterolemic and mildly hypercholesterolemic volunteers. The polyphenol ingestion, even in the lowest cocoa amount, was shown to contribute to the reduction in the plasmatic levels of LDL cholesterol, to the elevation of HDL cholesterol, and the suppression of oxidized LDL [189]. In another study, the ingestion of dark chocolate (100 g day^−1^ containing 70% of cocoa) for 7 days caused a significant increase in plasmatic HDL cholesterol concentrations and a non-significant decrease in LDL, total cholesterol concentrations, but showed favorable effects on inflammatory markers, confirming that polyphenol compounds from cocoa have contributed to beneficial effects on cholesterol metabolism [190]. 

Acute and sustained dose-dependent consumption of cocoa products have shown beneficial effects on endothelial function by FMD improvement [191], due to increases in plasmatic nitric oxide (NO) concentrations after cocoa beverage consumption in healthy subjects [192]. Cocoa polyphenols improve endothelial functions by increasing NO synthase (NOS) activity [168,169], leading to the inhibition of platelet adhesion and aggregation, and decrease of systolic and diastolic blood pressures [170].

## 7. Conclusions

Increasing evidence is available which indicates that the consumption of phenolic compounds present in natural foods may lower the risk of serious health disorders. The antioxidant power of polyphenols can effectively scavenge free radicals, absorb light in the UV region and chelate transition metals, among others. In addition to exerting protective effects, these compounds can also interact with intracellular signaling cascades, raising or activating transcriptional factors that impair the expression of several genes including, among others, COX_2_, _i_NOS, cyclins and NADP oxidase. Polyphenols can also act to regulate the activity of proteins and enzymes already synthetized in the organism. These molecular mechanisms, although not yet completely established, result in protection against chronic and degenerative diseases. 

The polyphenols found in root and tubercle vegetables, such as taro, beetroot and cassava, and in grains, like soybean and cocoa, described herein are involved in preventing chronic and degenerative diseases.

Regarding cardiovascular diseases, these polyphenols seems to be able to alter the lipid metabolism by inhibiting LDL oxidation, reducing atherosclerotic lesions, inhibiting platelet aggregation, decreasing malondialdehyde concentrations and, consequently, improving endothelial function and reducing systolic and diastolic blood pressures. In cancer, polyphenols are involved in inhibiting cancer cell proliferation and reducing the number of tumorigenic cells. In menopausal health impairments and symptoms, polyphenols, particularly the isoflavones-phytoestrogens found in soybeans, have been shown to reduce in vivolipid peroxidation and increase LDL resistance to oxidation, which in turncan improve bone metabolism, bone mineral density andcan promote cardiovascular protection, as well asmimic estrogen-hormones.

The food products described herein, in raw form, show a high diversity of phenolic compounds, but the lack of bioaccessibility for each form of consumption must be known before the recommendation of these products as dietary supplements or functional food ingredients for health promotion and/or reduction of risk diseases. Dosage recommendations and frequency of intake should be better evaluated. 

Moreover, natural polyphenols obtained from edible sources, by-products and co-products may display important applications in the food industry, where they can be used as dyes or food preservatives since they can arrest progressive food oxidation damage while avoiding the production of off-odors and off-flavors. Natural antioxidants from edible sources are considered safer alternatives for food preservation when compared to synthetic antioxidants, since they can avoid the adverse reactions of food additives to human health.

## Figures and Tables

**Table 1:** Flavonoid and non-flavonoid compounds plant sources.

Plant Source	Polyphenols	Class	Compound	Ref.
Beetroot (***B. vulgaris***)	flavonoids	flavanone	betagarin	[50]
		flavone	cochliophilin a	[50]
		flavonol	dihydroisorhamnetin	[50]
		isoflavone	betavulgarin	[50]
	non-flavonoids	hydrobenzoic acids	*n*-*trans*-ferruloylhomovanillylamine, *n*-*trans*-ferruloyltyramine	[50]
		hydroxycinnamic acids	caffeic acid, ferulic acid, gallic acid, *p*-cumaric, *p*-hydroxybenzoic, syringic acid, vanillic acid	[51]
Cassava (***M. esculanta***)	flavonoids	anthocyanidins	cyanidin, delphinidin	[52]
		flavan-3-ols	catechin, gallocatechin	[53]
		flavonols	kaempferol, quercetin, rutin	[54,55,56,57]
	non-flavonoids	coumarins	scopoletin	[58]
		hydrobenzoic acids	coniferaldehyde, gallic acid, isovanillin, syringsledehyde, resveratrol	[53,58]
		hydroxycinnamic acids	chlorogenic acid, *p*-coumaric acid	[53,58]
		lignans	balanophonin, pinoresinol	[58]
		stilbene	*trans*-3,3′,5,5′-tetrahydroxy-4′-methoxystilbene	[59]
Cocoa nibs (***T. cacao***)	flavonoids	anthocyanidins	arabinosidil, cyaniding, galactosidyl	[60,61]
		flavan-3-ols	cathechin, epicatechin, hyperoside, isovitexin, procyanidin b1, procyanidin b2, vitexin	[62]
		flavonols	quercetin, quercetin 3-*O*-arabinoside	[62]
		flavonones	apigenin, luteolin, luteolin-7-*O*-glucoside	[60]
		tannins	procyanidins	[60]
Soybean (***G. max***)	flavonoids	anthocyanidins	cyanidin, delphinidin, pelargonidin, petunidin	[63]
		hydrobenzoic acids	gallic acid, gentistic, protocatechuic acid	[64,65]
	non-flavonoids	hydroxycinnamic acids	caffeic acid, chlorogenic acid, ferulic acid, sinapic acid, *p*-coumaric acid, *t*-cinnamic acid	[65]
		isoflavonoids	β-glucosides: daidzin, genistin, glycitin malonyl-β-glucosides: malonyldaidzin, malonylgenistin, malonylglycitin acetyl-β-glucosides: acetyldaidzin, acetylgenistin, acetylglycitin aglycones: daidzein, glycitein	[64,65]
Taro (***C. esculenta***) (corms/leaves)	flavonoids	anthocyanidins	cyanidin, delphinidin	[66]
		flavonols	isorhamnetin, kaempferol, myricetin, quercetin	[66]
	non-flavonoids	hydroxycinnamic acids	chlorogenic acid, *p*-coumaric acid	[67]

**Table 2:** Human intervention studies evaluating the effects of polyphenols or polyphenol-enriched foods on health and diseases.

Polyphenols Source	Polyphenol Content(s)	Experimental Population	Number of Volunteers	Duration (Days)	Effect(s)
Isolated soy protein containing moderate and high isoflavones concentration	Two dietary groups: −56 mg of isoflavones −90 mg of isoflavones	Hypercholesterolemic postmenopausal women	66	168	Increases HDL cholesterol, mononuclear cell LDL receptor mRNA, both bone mineral content and density in the lumbar spine after ingestion of two dietary groups decreases in non-HDL cholesterol after ingestion of the two dietary groups (56 and 90 mg of isoflavones) [164].
Genistein (soy phytoestrogen)	54 mg	Healthy and postmenopausal women (range 47–57 years)	90	364	Decreased excretion of pyridinium and deoxypyridinoline (PYR: −54 ± 10%; DPYR: −55 ± 13%) after 6 and 12 (PYR: −42 ± 12%; DPYR: −44 ± 16%) months of genistein administration. Increases in serum bone-specific ALP (B-ALP) and osteocalcin (bone Gla protein [BGP]) after 6 (B-ALP: 23 ± 4%; BGP: 29 ± 11%) and 12 (B-ALP: 25 ± 7%; BGP: 37 ± 16%) months of genistein administration. Furthermore, significantly increases in femur (femoral neck: 3.6 ± 3% and lumbar spine (3 ± 2%) bone mineral density (BMD) were observed [165].
Textured soy protein high in isoflavones (HI); Textured soy protein low in isoflavones (LI)	HI 21.2 mg of daidzein; 34.8 mg of genistein; LI 0.9 mg of daidzein 1.0 mg of genistein	Healthy men and women (range 19–40 years)	24	14	Decreased plasma 8-epi-PGF_2_α after high-isoflavone dietary treatment (326 ± 32 ng L^−1^) when compared to the low-isoflavone dietary treatment (405 ± 50 ng L^−1^). The lag time for copper-ion-induced LDL oxidation was longer after high-isoflavone dietary treatment (48 ± 2.4 min) than low-isoflavone dietary treatment (44 ± 1.9 min). No changes in plasma malondialdehyde, LDL α-tocopherol, polyunsaturated fatty acids, and isoflavonoids after dietary treatments [166].
Cocoa supplementation (dark chocolate bar and cocoa powder drink)	651 mg of procyanidins	Healthy men and women (range 20 to 60 years)	25	42	Decreased LDL oxidizability (evidenced by a longer lag time, 101.0 ± 20.7 min) after cocoa supplementation compared with baseline (91.3 ± 18.0 min) and washout (96.4 ± 7.5 min). No changes in urinary F(2) isoprostane concentration and markers of inflammation including the whole-blood cytokines, interleukin-1 beta, interleukin-6 and tumor necrosis factor-alpha, high sensitivity C-reactive protein and P-selectin [167].
Cocoa drink	821 mg of total flavonoids	Healthy men and women (range 18–72 years)	27	35	Increased peripheral vasodilation after four days of cocoa drink ingestion. After five days of cocoa drink consumption, pulse wave amplitude exhibited a large additional acute response [168].
Cocoa drink	176 mg of flavan-3-ols (70 mg of epicatechin plus catechin and 106 mg of procyanidins)	Outpatients with at least 1 cardiovascular risk factor (means, 41 years)	26	2	Increased flow-mediated dilatation maximally at 2 h from 3.4% to 6.3% after cocoa drink ingestion. Increases nitrosylated and nitrosated species from 22 to 36 nmol L^−1^ after ingestion of cocoa rich in flavan-3-ols [169].
Dark chocolate	15.6 mg of epicatechin equivalents per gram	Heart transplant recipients volunteers (range 35–70 years)	22	Acute	Increased coronary artery diameter from 2.36 ± 0.51 to 2.51 ± 0.59 mm after ingestion of flavonoid-rich dark chocolate. Decreased platelet adhesion from 4.9 ± 1.1% to 3.8 ± 0.8% after ingestion of flavonoid-rich dark chocolate [170].
Powder cocoa drink	963 mg of flavonoids	Diabetes mellitus II men and women (for at least 5 years, range 50 to 80 years)	41	28	Increased flow-mediated dilatation (FMD) by 30% after ingestion of flavanol-containing cocoa. Treatment was well tolerated, without evidence of tachyphylaxia. No changes in endothelium-independent responses, blood pressure, heart rate, and glycemic control after ingestion of the cocoa drink [171].
